# Miscibility and thermal behavior of poly(methyl methacrylate) and polystyrene blend using benzene as a common solvent

**DOI:** 10.55730/1300-0527.3498

**Published:** 2022-09-16

**Authors:** Arshiya IQBAL, Jamil MUHAMMAD, Farzana AHMAD, Mohsan NAWAZ, Musa Kaleem BALOCH, Luqman MOHAMMAD

**Affiliations:** 1 Institute of Chemical Science, Department of Chemistry, Gomal University, Dera Ismail Khan, Pakistan; 2 Department of Chemical and Life Sciences, Qurtuba University of Science & Information Technology, Dera Ismail Khan, Pakistan; 3 Sang-Ho College & Department of Physics, Konkuk University, Seoul, Korea; 4 Department of Chemistry, Konkuk University, Seoul, Korea; 5 Department of Chemistry, Hazara University, Mansehra, Khyber Pakhtunkhwa, Pakistan; 6 Chemical Engineering Department, College of Engineering, Taibah University, Yanbu Al-Bahr, Kingdom of Saudi Arabia

**Keywords:** Miscible, immiscible, polymer solutions, compatibility, polymer blends, viscosity, thermal stability, flow activation energy, polystyrene (PS), poly(methyl methacrylate) **(**PMMA)

## Abstract

Polymer blending is one of the advanced technologies to attain polymeric material with tailored properties. In this work, the miscibility of Poly(methyl methacrylate) (PMMA) and Polystyrene (PS) blend in benzene was investigated by employing various techniques such as FTIR spectroscopy, viscosity measurement technique, light scattering techniques, DSC and TGA techniques over an extended range of concentrations, compositions, and temperatures. The results revealed that there exist hydrogen bonding and hydrodynamic interactions which led these polymers to get miscible to a large extent. The compatibility increased with the increasing PS contents or increase in temperature of the system. In addition, the thermal stability of blends was found to be improved with the increase in the compatibility of the polymer.

## 1. Introduction

Polymer blending technology is considered to be cost-effective, and easier to get the material of required properties to a large extent. However, the preparation and processing of new polymer blends and the control of their morphology require a comprehensive knowledge of the thermodynamics of polymer mixture [1–7]. In the past much intensive research has been performed for understanding the characteristics of polymer blends, both miscible and immiscible. Due to the decreasing entropy of mixing for high molecular weight chains, miscibility is unusual. As a result, the free energy balance for systems forming one-phase liquids at high molecular weights is often subtle [1]. It is well known that favourable interactions are a prerequisite for miscibility in hetero-polymer blends because of the very small entropy of mixing in high-molecular-weight polymer mixtures. The heat of mixing (ΔH_m_) of a polymer system then becomes a direct measure of favorable interactions. There are, however, experimental obstacles that prevent the direct measurement of ΔH_m_. For example, the high viscosity of polymer systems retards attainment of equilibrium; also there can be T_g_ intervention which affects the mixing–demixing interactions [1–7]. However, such issues can be handled by using the low-molecular-weight analogue method via measuring ΔH_m_. Another possible approach is by employing Hess’ law for the estimation of experimental enthalpies of solutions for the blend and pure components. A similar but somewhat more favourable situation exists in the theoretical treatment of the thermodynamics of polymer blends. The equation derived from mean-field theories, which are extensions of the classical Flory Huggins’ (FH) theory, can be fitted to experimental data to find the phenomenological interaction parameter χ. This parameter represents an average overall interactions and, to a large extent, absorbs other effects such as those associated with the equation of state, composition, and chain length. The problem is that FH theories assume random mixing and do not account for correlations in chains that is chain connectivity. Derivations from random mixing are for the most favourable interactions proposed up to now [1–7].

Market size of the polymer alloys and blends during 2020 to 2025 is forecasted to reach almost $5.3 billion dollar by 2025 with a growth rate at a compound annual growth rate (CAGR) of 5.40% [8]. Despite the progress in the blending technology, there is still a need to improve the technology about the prediction of blends’ thermal/mechanical properties which correlates the structure of polymers blended and their miscibility/compatibility [9–13]. Therefore, many scientists are working over the establishment of techniques through which the miscibility/compatibility of polymers in a blend can be estimated and then correlated to the thermal and mechanical properties of blends [14–22,58]. Among these techniques, viscosity measurement is considered to be simple, easy to measure and provides reasonably good results [23,24].

Among various industrially important polymer blends, the blends of poly(methyl methacrylate)-PMMA and polystyrene-PS form a very unique and important pair of polymers. PMMA is a thermoplastic polymer and is well known and widely used industrial plastic for its excellent optical properties. It is used as organic glass as a replacement for inorganic glasses. It is light in weight, flintier, stiff, colourless, transparent, have good weather/chemical resistance and offers good insulation. However, it is brittle in pure form, limiting its wider use. PS is also one of the most widely used industrial and domestic polymers. It is inexpensive, clear, hard, and brittle. These polymers are being combined together to get benefitted from each other’s drawbacks.

A few studies have been conducted to investigate the chemical, thermal, mechanical, viscous, and morphological behaviour of this polymer pair. Thermal parameters of blends are very important aspects to be studied from an industrial point of view. The model blend of this pair is reported by a few researchers; however, clear information still needs to be provided regarding the thermal parameters of this blend [25] and references therein [17–22]. Additionally, the intermolecular chemical interactions between two or more different polymer chains in solutions play an active role in understanding viscosity behaviours. A few studies claim that this pair is incompatible or immiscible in all compositions studied, however, in contrast, a few other studies show that this pair is partially compatible with certain compositions and/or solvents. For example, Sun and Wang [26] concluded through quasi-elastic light scattering studies that the interaction between their chains in the benzene solution was repulsive. Khan et al. [27] through dilute solution viscometry and FTIR techniques concluded that this pair in tetrahydrofuran (THF) solution was immiscible. A recent study in 2019 by Akpan et al. [54] also observed through FTIR and viscometry studies that this pair showed no compatibility in chloroform solution. Mathur and Sharma [28] concluded that the phase interactions between this polymer pair in THF are physical, not chemical. The existence of two well-defined glass transition (T_g_) peaks related to PS and PMMA phases, respectively, these blends over the studied composition range revealed that this pair of polymeric blends are highly immiscible. Interestingly, in contrast, Melad et al. [29] found slight compatibility of these polymers at all concentrations in dimethylformamide (DMF) with increasing compatibility with increasing PMMA concentration, but no compatibility in THF. In a recent study in 2019, Hameed et al. [30] through UV-Vis spectrophotometric and optical microscopic studies concluded that in this PS/PMMA pair in dichloromethane solution was found to be well compatible up to 40 wt% of PS concentration, however, there was a very visible phase separation above this concentration. Based on this literature review, it is clear that further studies are still needed to understand the subject well. Therefore, we have applied the viscosity measurement technique to estimate the compatibility of PS and PMMA dissolved in benzene and verified it using FTIR and DSC and investigated the impact of miscibility against thermal stability by using thermal degradation. The objective of the current work was to prepare blends of various polymers and explore the impact of miscibility of these polymers over their degradation mechanism, melting process, Tg, and mechanical behaviour of the blend and correlate the miscibility of polymers with mechanical and thermal properties of the blend.

## 2. Theoretical background

Dilute solution viscosity measurement (DSV) uses classical Huggins equation [31,32] which is expressed as under;


(1) $$\frac{\eta_{sp}}{C}=[\eta ]+bC$$

(2) $$\text{b}={\text{K}}_{\text{H}}\;{\left[\eta \right]}^{2}$$

Where η_sp_ and [η] stand for specific viscosity and intrinsic viscosity of solution, respectively [33]. Further, b and C represent polymer-solvent binary interactions, and mass concentration of solution, respectively [33], and the k denotes Huggins coefficient which represents binary interactions between solvent molecules and polymer segments.

### 2.1 Viscosity interaction parameters

We have used following approaches to calculate the viscosity interaction parameters of polymer-solvent and polymer (1)-polymer(2)-solvent based binary and ternary systems, respectively [34].

Garcia et al. [35] conducted miscibility studies of polymer blend solution using Δ[η]_m_;


(3) $$\Delta {\left[\eta \right]}_{m}={\left[\eta \right]}^{exp}-{\left[\eta \right]}^{Theo}$$

Where


(4) $${\left[\eta \right]}^{Theo}=w_{1}\;{\left[\eta \right]}_{1}+w_{2}\;{\left[\eta \right]}_{2}$$

Δ[η]_m_ < 0 shows miscibility of the blend, and vice-versa.

After modifying the classical Huggins equation, Krigbaum–Wall [36] applied it to the ternary system.

The following methodology was proposed for the determination of the miscibility of polymer blends;


(5) $$\frac{{\left(\eta_{sp}\right)}_{m}}{C_{m}}={\left[\eta \right]}_{m}+b_{m}\;C_{m}$$

Where (η_sp_)_m_ is the specific viscosity, C_m_ is the total concentration of component polymers C_1_+C_2_, and b_m_ is the global interaction between polymer components of the blend [33];


(6) $$b_{m}=w_{1}^{2}b_{11}+w_{2}^{2}\;b_{22}+2w_{1}\;w_{2}\;b_{12}^{exp}$$

Where b_m,_ b_11_, b_22_ are the interaction parameters of the polymer blend, polymer 1 and polymer 2, respectively [33], that are obtained from the slopes of the plots of reduced viscosity versus concentration. While is the interaction parameter of the blend calculated [33]; from Equation (6), w_1_ and w_2_ are the weight fractions of polymers 1 and 2, respectively [33–35].

The following criteria was suggested by Chee [38] to estimate polymer-polymer miscibility in solution;


(7) $$\Delta B=\frac{b-b^{-}}{2w1w2}$$

Where b̄ = w_1_b_11_+w_2_b_22_, in which w_1_ and w_2_ are weight fractions, and b_11_ and b_22_ are the slopes of the viscosity plots for the pure polymers [33]. For a ternary system, ^‘^b^’^ can be expressed as [33];


(8) $$b=w_{1}^{2}\;b_{11}+w_{2}^{2}\;b_{22}+2\;w_{1}w_{2}b_{12}$$

Where b_12_ is the slope of the viscosity plot for the solution of the blend.

ΔB ≥ 0 reflects the miscibility of polymer blend and vice-versa. If the intrinsic viscosities of pure polymer solutions are very different [33], the Chees’s approach does not take into account for the experimental data. Thus, in this case, Chee, identified a more reliable and effective parameter for the estimation of polymer miscibility;


(9) $$\mu =\frac{\Delta B}{\left[\eta_{1}-\eta_{2}\right]}$$

Where [η]_1_ and [η]_2_ are the intrinsic viscosities for pure polymers. μ < 0 shows immiscibility, while μ ≥ 0 signifies miscibility of polymers in the blend [33].

Sun et al. [39] also proposed the following parameter () to estimate the miscibility of polymer blends;


(10) $$\alpha =k_{m}-\frac{{\left[(\sqrt{k_{1}})\eta_{1}w_{1}+({\sqrt{k}}_{2})\eta_{2}\;w_{2}\right]}^{2}}{{\left[\eta_{1}w_{1}+\eta_{2}\;w_{2}\right]}^{2}}$$

Where $k_{1}=\frac{b_{11}}{{\left[\eta \right]}_{1}^{2}},\;k_{2}=\frac{b_{22}}{{\left[\eta \right]}_{2}^{2}}$ and $k_{m}=\frac{b_{m}}{{\left[\eta \right]}_{m}^{2}}$

α ≥0 reflects mutual compatibility and/or attraction between individual polymers in solution [34], and hence, miscibility while α < 0 reflects mutual incompatibility and/or repellency, and hence, immiscibility of polymers in the blend [34,1].

Jiang and Han [40], however, replaced α with β as an improved parameter as shown below;


(11) $$\beta =\frac{2\Delta kw_{1}\;w_{2}{\left[\eta \right]}_{1}{\left[\eta \right]}_{2}}{{\left[w_{1}\eta_{1}+w_{1}\eta_{2}\right]}^{2}}$$

Here *Δk* = *k*_12_ – (*k*_1_ *k*_2_)^1/2^ and $k_{12}=\frac{b_{12}}{{\left[\eta \right]}_{1}\;{\left[\eta \right]}_{2}}$

## 3. Experimental

### 3.1. Materials

PS and PMMA, supplied by Shell polymers, were used. Some of the basic features and commercial sources of the polystyrenes (PS), and poly(methyl methacrylate) (PMMA) are employed in this work. Their basic details are mentioned elsewhere (in Table 1) [55]. By employing the blends of these two polymers we have investigated the miscibility, thermal stability, and morphology of the PS/PMMA blends [55]. The benzene (of analytical grade) was used as a solvent and was obtained from Fluka, Germany. It was used as such without further purification.

### 3.2 Preparation of polymer stock solutions

Solutions of PS and PMMA were prepared in benzene separately by dissolving 25 g of polymer in a 1000 mL volumetric flask. For this purpose, the calculated amount of the polymer was transferred into the volumetric flask, and a small volume of benzene was added. Polymer-solvent mixtures were kept for 24 h to facilitate the full dissolution of polymers. Then additional volumes of solvent were transferred into the flask to make the total volume of the solution up to one L. The required concentration of polymers was obtained through the dilution method.

### 3.3. Preparation of blends

Blends having 30/70, 50/50, and 70/30 compositions of PS/PMMA were prepared by mixing required volumes of stock solutions of PS and PMMA.

### 3.4. Preparation of films

The solutions of blends were cast onto a glass plate and were dried at 25 *°*C for 24 h and dried further in an oven at 80 *°*C to remove traces of solvent. This resulted in solvent-free and mechanically stable films.

### 3.5. Viscosity measurement

Ostwald’s capillary viscometer was used for measuring dilute solution viscosity (DSV) of PS and PMMA and their blends of various ratios at different (20–50 *°*C) temperatures. The viscometer was thoroughly cleaned with the chromic acid mixture, followed by distilled water, and then was dried for each measurement. The system was thermostated by using a water thermostat supplied by F.G. BOODE and CO, Germany, up to one decimal point of a degree celcius [33]. The results obtained were used for the estimation of intrinsic viscosity using Huggins’ Equation [31, 32] (Equation (12)):


(12) $$\frac{\eta_{sp}}{C}=[\eta ]+K_{H}\;{\left[\eta \right]}^{2}C$$

Here ɳ_sp_, K_H_, c and [η] are the specific viscosity, Huggins’ constant, concentration of polymer and intrinsic viscosity of the polymer, respectively.

The molecular mass of the polymer was obtained by employing the Mark-Houwink equation [41–43] [Eq. (13)].


(13) $$[\eta ]={\text{KM}}^{\text{a}}$$

Here M is the molecular mass of the polymer, K and a are the Mark–Houwink equation (MHE) constants of the system.

### 3.6. Light scattering measurement

The laser light scattering (LLS) measurements were performed for polymers of different concentrations at a constant temperature [33]. The instrument which was used for this measurement was DAWN EOS, Wyatt, USA. A cylindrical cell (SV) of 2 cm diameter and the Helium-neon laser of 632.8 nm wavelength were used as a light source for this measurement [33]. Before this measurement, samples were passed through filters of 0.02 μm and 0.25 μm pore sizes for the solvent (benzene) and solution, respectively.

The following equation was used for the analysis of the results:


$$\frac{KC}{R_{\text{q}}}=\left[1+\frac{16{\text{p}}^{2}n^{2}-R_{g}^{2}-{\text{Sin}}^{2}(\text{q}/2)}{{3|}^{2}}\right]\left[\frac{1}{M}+2A_{2}C\right]$$

and it can be simplified to Eq. (13) when θ approaches to zero


(14) $$\frac{Kc}{R_{\theta }}=\frac{1}{M_{w}}+2A_{2}c$$

R_θ_ is the Rayleigh ratio, θ is the angle of measurement, n is the refractive index of the solvent, and λ is the wavelength of laser light. A_2_ is the second virial coefficient and K is defined by Eq. (15).


(15) $$K=\frac{4{\text{p}}^{2}n^{2}{\left(dn/dC\right)}^{2}}{N_{A}{|}^{4}}$$

The (dn/dC) is the refractive index increment of the polymer solution, while N_A_ is Avogadro’s constant. The intercept of a plot of KC/R_θ_ versus C will give 1/M_w_ and hence molecular mass was obtained.

### 3.7. FTIR measurement

FTIR spectra of dried films of PS-PMMA polymer blends having a different ratio of polymers were obtained at constant temperature for the whole available range of wavenumbers, using FTIR Spectrometer, Tensor 27 (Bruker, Germany).

### 3.8. Differential scanning calorimetry (DSC)

For the purpose, Diamond DSC Pyris-1 supplied by Perkin-Elmer, USA was used and the measurements were carried out under the nitrogen atmosphere (flow rate of 80 mL. min^−1^. nitrogen). Polymer samples of ca. 7 mg were placed in an aluminium pan and the DSC thermograms were obtained by heating the system at a rate of 5 *°*C/min up to 280 *°*C.

### 3.9. Thermo-gravimetric analysis (TGA)

The TGA of PS, PMMA, and their blends were performed using Diamond Thermo-gravimetric/ Differential Thermal analyzer (TG/DTA), Perkin Elmer, USA [33]. Dry polymer samples, whose weight was ca. 3 mg, were put into the aluminium pan. In order to maximize the physical contact between the pan and sample, a lid was crimped [33]. The polymer samples were heated at a constant rate of 5 °C/min from room temperature to 600 °C under nitrogen purge. Using this technique, one can get the onset temperature (the temperature at which thermal degradation starts), the number of steps in which thermal degradation takes place (may be single, double, triple etc), time taken by the sample for thermal degradation and the residue of the material. On the other hand, the rate of thermal degradation can be estimated for a particular temperature or at the temperature at which the degradation rate is maximum from the slope of the TGA curve (weight loss/unit time for a particular temperature.

## 4. Results and discussion

**Figure 1:**
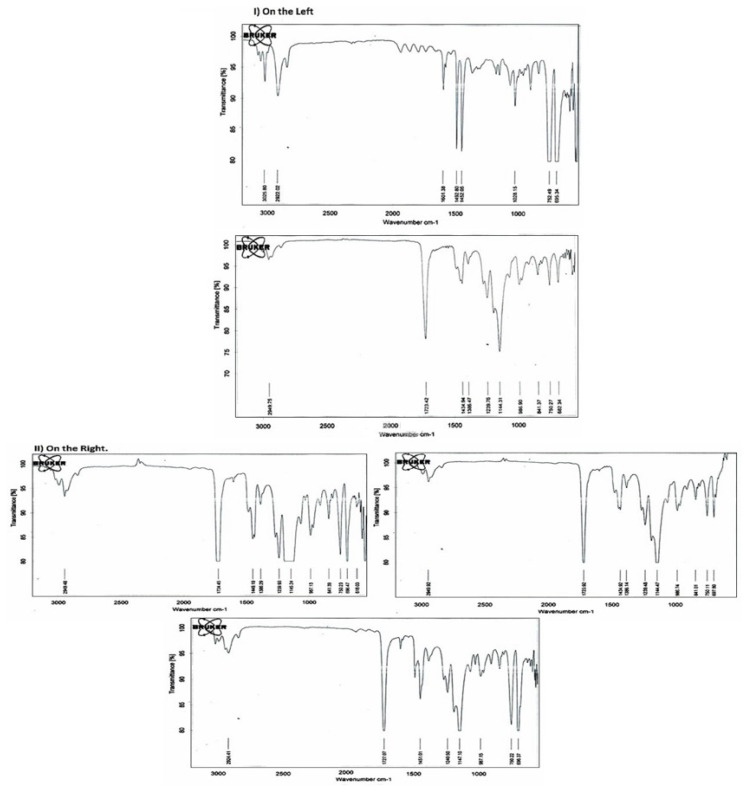
On the left) FTIR spectra of (a) polystyrene and (b) polymethylmethacrylate (PMMA), II. On the right) FTIR spectra of PS/PMMA blend.

The FTIR results of the polymers were compared with the library of the instrument/literature and the material investigated was confirmed to be polystyrene and poly (methyl methacrylate) (Figure 1). Briefly, pure PMMA displayed four clear absorption bands at 2949.75 cm^−1,^ 1723.42 cm^−1^, 1144.31 cm^−1^ and 1239 cm^−1^ and were attributed to C-H stretching [33] [44–45]; C=O of the ester group of PMMA [44–47]; −O-CH_3_ stretching of the ester group [45–47], CH_3_ stretching [45–47]; respectively. In the fingerprint area of the IR spectrum, the peak at 749 cm^−1^ corresponded to out of plane C-H bending [33].

In the case of pure polystyrene, the absorption bands which are visible at 3025.80 cm^−1^ represent C-H stretching. The presence of the benzene ring of polystyrene can be identified from two regions ([33], 1) the broad absorption band near 3025 cm^−1^ owing to C-H stretching, 2) the absorption band consisting of a series of four peaks in the region of 1670 cm^−1^ is the characteristics of the conjugation bonds [33], together with concluding the presence of polystyrene. To verify the results, we have measured the FTIR of the film performed from the pure polymers and their blends. Some of the spectra obtained for the blends are displayed in Figure 9. FTIR spectra of polymer blends having a varying percentage of PS and PMMA show the absorption bands that are characteristics of both polymers [33]. The characteristics peaks of virgin PMMA; 1723.42, 1434.94, 1239.76, 1144.31, 986.90 were shifted to 1724.45, 1449.19, and 987.13 in case of 70/30 PS/PMMA blend [33], and to 1727.07, 1451.01, 1240.50, 1147.10 and 987.15 in case of 50/50 PS/PMMA, which indicates that polymers in these two blends are partially miscible [33], [48–49]. However, there was no shifting in peaks in the case of the 30/70 PS/PMMA blend, which showed that these polymers are not miscible and got phase separated. This data is validated by similar results from the viscosity data.

**Figure 2:**
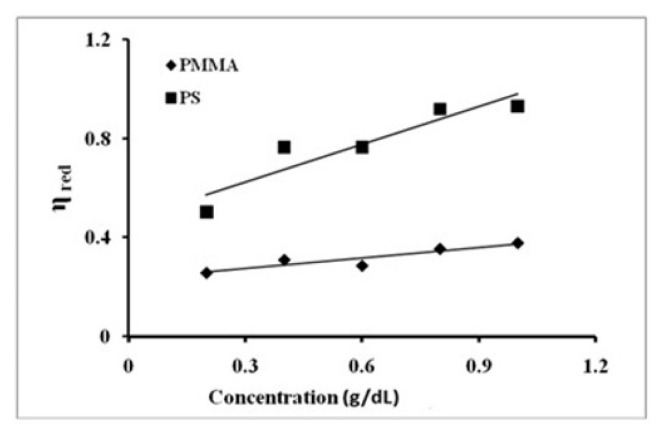
Reduced viscosity as a function of concentration of PMMA and PS at 30 °C.

**Figure 3:**
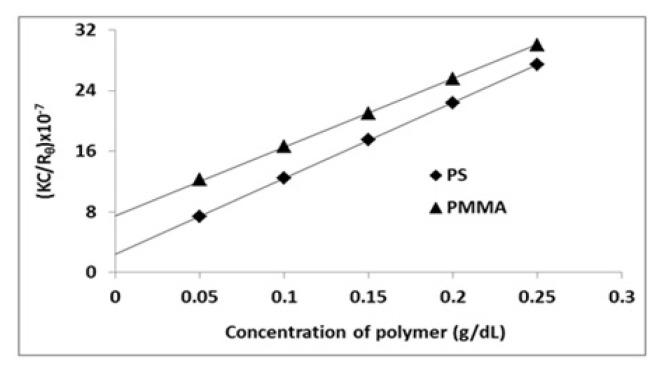
Kc/R*_θ_* values of PS and PMMA measured in benzene.

To estimate the molecular weight of the polymers, the reduced viscosity was plotted versus the concentration of polymer according to Equation (12) and the intrinsic viscosity was obtained from the intercept of these plots Figure 2. From the intrinsic viscosity, the molecular weight of the polymers was obtained using Equation (13). The molecular weight obtained in this way was ca. 1.3 × 10^6^ and ca. 4.1 × 10^6^ g/mol for PMMA and PS, respectively. The molecular weight of polymers was also obtained by the light scattering technique by plotting Kc/R*_θ_* versus c as per Equation (14). The intercept of the plots provided molecular mass as 4.08 × 10^6^ and 1.33x10^6^ g/mol for PS and PMMA Figure 3, which were higher than the one obtained by viscosity measurement, indicating that the degree of dispersity was greater than one. The second virial coefficient was obtained as 4.5 *×* 10^−6^ and 2.5 *×* 10^−6^ for PS and PMMA, respectively.

**Figure 4:**
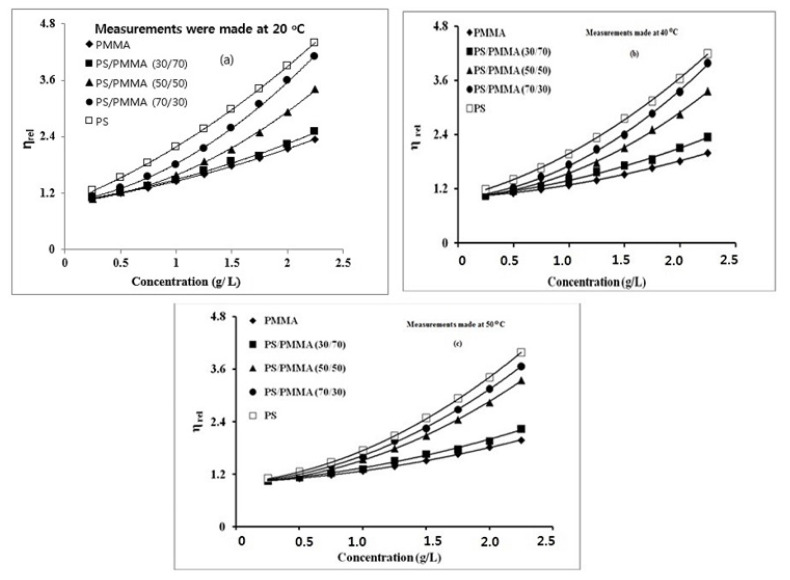
Relative viscosity as a function of concentration of PS/PMMA blends measured at (a) 25 °C, 40 °C, and (c) 50 °C.

Polymers were dissolved in benzene and their blends with the composition PS^_^PMMA (30/70), (50/50), and (70/30) were prepared. The solution viscosity was measured at 20, 30, 40, and 50 *°*C. The relative viscosity of polymers blends solution was calculated and plotted against their composition and their concentration in Figure 4. The data displayed linear plots up to certain concentrations and then became nonlinear. The concentration at which the plots became nonlinear decreased with the increasing amount of PS. This trend was explained in terms of the molecular mass of the polymers; the higher the molecular mass, the lower concentration was needed to set in the molecular interactions. The trend remained almost constant and was independent of the temperature of the system. The only notable difference was in the viscosity which decreased with increasing temperature. Though such an observation is considered to be a characteristic of a miscible blend system but is hard to conclude from such curves [50–52], [32].

**Figure 5:**
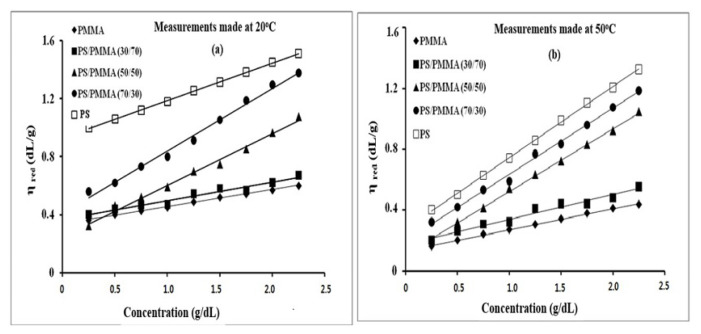
Reduced viscosity as a function of concentration of PS/PMMA blends measured at (a) 20 °C and (b) 50 °C.

The reduced viscosity (*η*_sp_/c) with respect to C for these pure polymers and their blends in benzene solvent, obtained at different temperatures were plotted. All the plots were found to be linear over the measured range of concentrations and temperatures mentioned in Figure 5. The figure displayed that the viscosity was decreasing with increasing temperature, matching well with our expectations.

**Figure 6:**
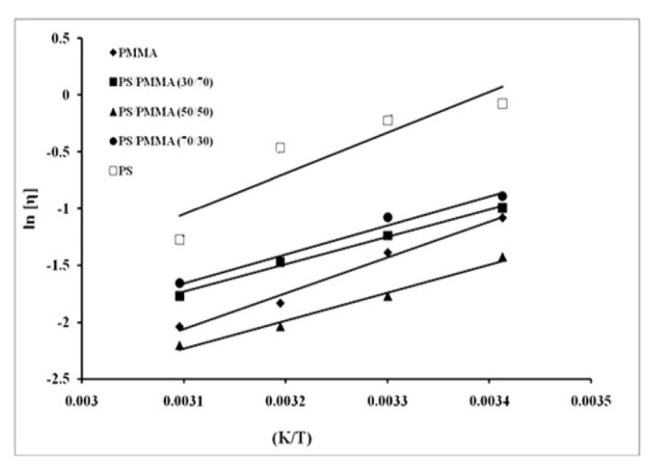
Log of intrinsic viscosity of PS/PMMA blends as a function of temperature.

The intrinsic viscosity as per Equation (1) can be calculated from the intercept of the plots [51]. These figures also indicated that intrinsic viscosity is not in accord with the composition of the blend. For example, intrinsic viscosity of PS/PMMA (50/50) blend measured at 25 °C was less than the 30/70 or the 70/30 blend and the difference was decreased with the increase in temperature of the system. From these observations, we can conclude that the miscibility increased with increasing temperature. The intrinsic viscosity based on the obtained data vs. temperature was plotted in Figure 6. It can be seen that it decreased with increasing temperature in correlation with Equation (16).


(16) $$\text{ln}[h]=\text{ln }k-\frac{E_{a}}{RT}$$

**Figure 7:**
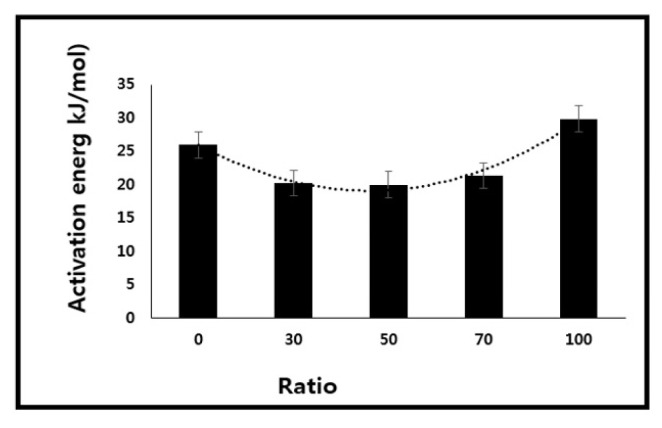
Flow activation energy of blends having different ratios of PS/PMMA.

Here E_a_, R, and K are the flow activation energy [57] of the system, gas constant, and preexponential factor, respectively. The E_a_ of pure polymers and their blends was obtained using Equation (16) and presented in Figure 7. It was found that the blending reduced the flow activation energy [57] of the polymers. Thus, it may be suitable to conclude that the polymer-polymer interactions were lower as compared to those of polymer-solvent interactions, and hence, required higher energy to flow. This also means that molecular interactions were reduced by mixing the two polymers as compared to a single polymer-solvent system which is certainly an indication of the immiscibility of polymers [1].

Let us suppose that the slope of reduced viscosity-concentration curves is equal to b. While b can be then defined as per Equation (17). In the following equation, b represents the binary interactions between the polymer chains.


(17) $$\text{b}={\text{K}}_{\text{H}}\;{\left[\eta \right]}^{2}$$

For ternary (polymer1/polymer2/solvent) system Krigbaum and Wall [36] proposed an equation analogous to Equation (12), which is displayed as Equation (18).


(18) $$\frac{{\left(\eta_{\text{sp}}\right)}_{\text{m}}}{{\text{C}}_{\text{m}}}={\left[\eta \right]}_{\text{m}}+{\text{b}}_{\text{m}}\;{\text{C}}_{\text{m}}$$

Here (*η*_sp_)_m_ represents the specific viscosity of blend, C_m_ (= C_1_ and C_2_) is the sum of concentration of polymers, is the intrinsic viscosity of the blend and b_m_ signifies the global interaction among all polymeric species, which is represented by Equation (19).


(19) $$b_{m}=X_{1}^{2}b_{11}+2X_{1}X_{2}b_{12}+X_{2}^{2}b_{22}$$

Here X_1_ and X_2_ are the weight fractions of polymer 1 and polymer 2, respectively [34]. b_11_ and b_22_ are the interaction parameters for polymer 1 and polymer 2, and b_12_ is the interaction parameter for the blend system [33] which can be calculated from Equation (19). The interaction parameters b_11_, b_22_ and b_m_, were calculated [51], [36] from the slopes of the plot of reduced viscosity vs. concentration (Table 1), and b*_12_ was obtained, theoretically, using Equation (20).


(20) $$b_{12}^{*}={\left(b_{11}b_{22}\right)}^{1/2}$$

The obtained results illustrated that polystyrene showed the highest level of intrinsic viscosity, and it decreased with decreasing amount of polystyrene in the blend, which was found to be independent of the temperature. It was also observed that their slope (K_H_ [***η***]^2^) increased with an increasing amount of PS and/or temperature. The slopes of the plots for pure polymers, however, were lower than those of the blend. The difference in b values (=*Δ* b) was calculated from the theoretical b^*^_12_ using Equation (20) and the experiment, b_12_ was obtained by using Eq. (19) and Krigbaum and Wall [36] equation Equation (21) and viscosity results.


(21) $$\Delta b=(b_{12}-2b_{12}^{*})$$

For Δb < 0, phase separation occurs or blend is treated immiscible, while at *Δ* b > 0, blends are treated miscible. It has been found that Δ b values are positive (Table 2) for the 70/30 and the 50/50 indicating miscibility and for the 30/70 values are negative showing immiscibility. The same table contains the respective Δk_AB_ as well.

**Figure 8:**
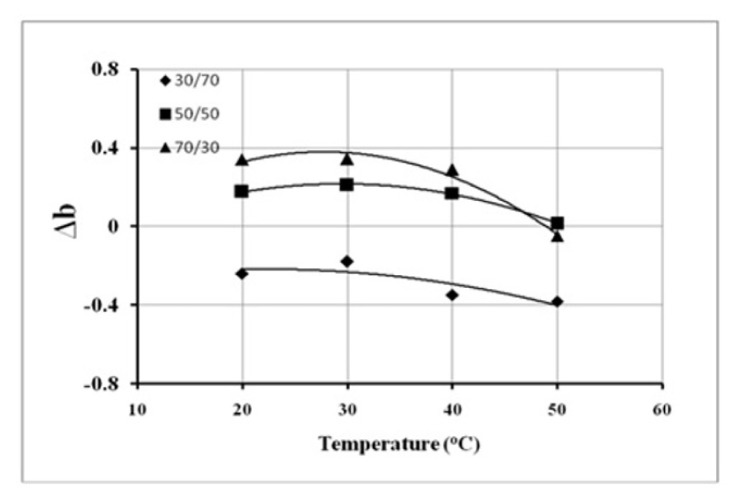
Variations in Δb values of PS/PMMA blends as a function of temperature.

According to Krigbaum and Wall [36], *Δ*b > 0 indicates that the polymers in the blend are miscible and vice-versa. The values of Δb obtained in this way are plotted in Figure 8, as a function of temperature. It can be seen that irrespective of the polymer ratio in the blend, the miscibility of polymers increased up to 30 *°*C followed by a sharp decrease with increasing temperature. The overall miscibility, however, was found to be the highest for the 70/30 sample, and the lowest for the 30/70 PS/PMMA blend. It means that PS can be solubilized in PMMA. Here Figure 8, further indicated that the 30/70 blend was incompatible at all the investigated temperatures, whereas the 70/30 was incompatible only for 50 *°*C.

If η_1_ and η_2_ are significantly different, Chee [38] defined a more effective parameter μ, which can be used to determine the compatibility between the constituents of the blend, for which, the equation is as follows:


(22) $$\mu =\Delta \text{b}/{\left(\eta_{2}\;\eta_{1}\right)}^{2}$$

where η_1_ and *η*_2_ are intrinsic viscosities of pure individual polymer solutions. The blend is treated immiscible if μ < 0, and is miscible when μ > 0. Table 3 presents the calculated values of μ as well as values of *α*. The results showed that the μ are positive for 70/30 and 50/50, but 30/70 ratio showed a negative value indicating system in this ratio is immiscible.

Sun et al. [39], suggested a new way to determine the miscibility of polymers as follows;


(23) $$\text{a}=K_{m}-\frac{K_{A}{\left[\text{h}\right]}_{A}^{2}W_{A}^{2}+K_{B}{\left[\text{h}\right]}_{B}^{2}W_{B}^{2}+2{\sqrt{K_{A}K_{B}[\text{h}]}}_{A}{\left[\text{h}\right]}_{B}W_{A}W_{B}}{{\left({\left[\text{h}\right]}_{A}W_{B}+{\left[\text{h}\right]}_{A}W_{B}\right)}^{2}}$$

**Figure 9:**
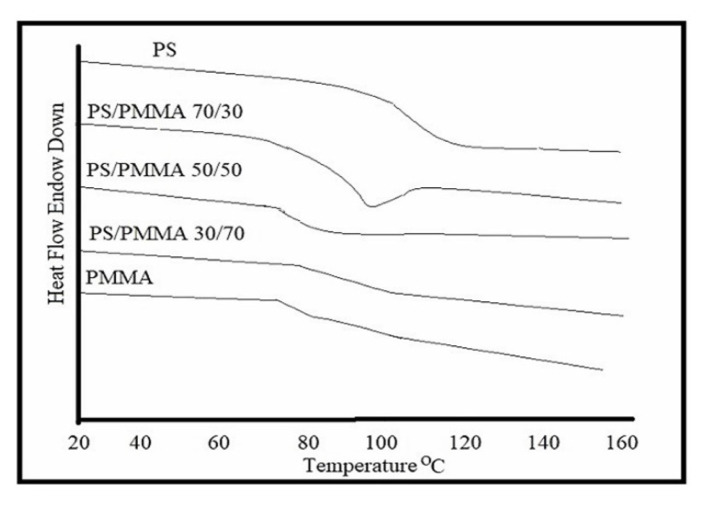
DSC curve of PMMA and PS and their blend.

**Figure 10:**
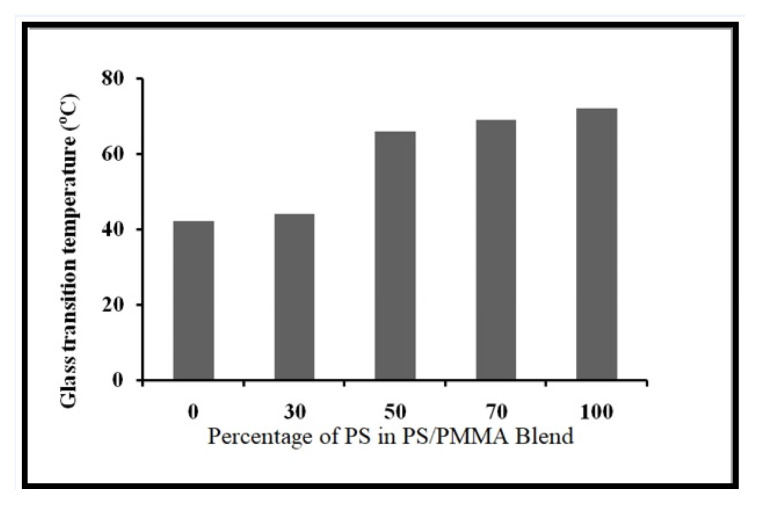
Glass transition temperature of PS/PMMA blend.

**Figure 11:**
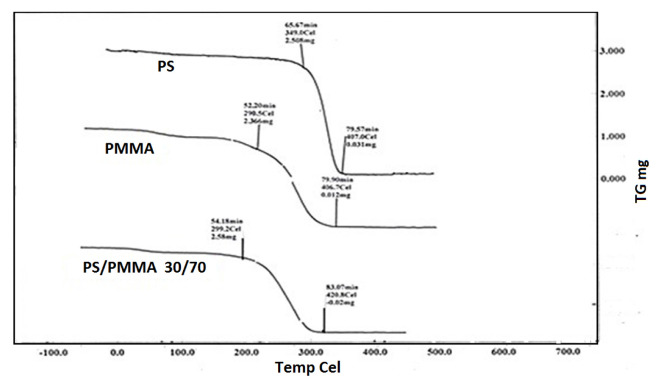
TG curve of PS, PMMA, and PS/ PMMA 30:70 blend; heating rate of 5 *°*C/m.

**Figure 12:**
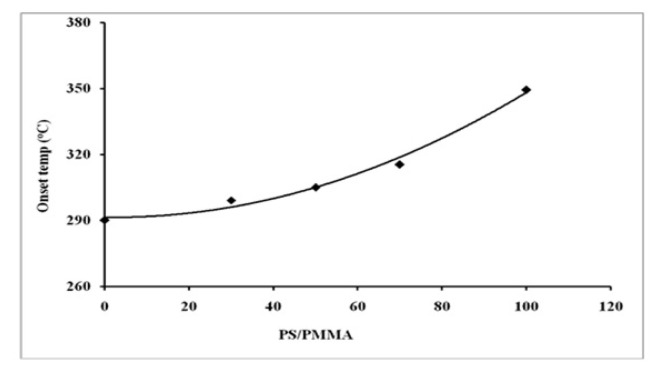
Onset temperature of blend as a function of blend ratio.

**Figure 13:**
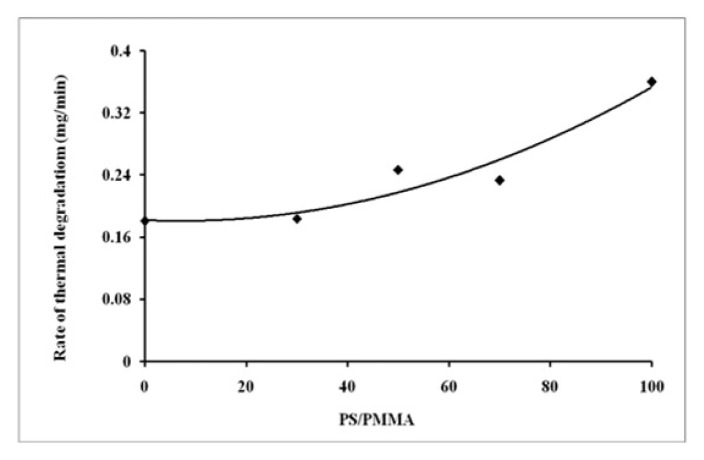
Rate of degradation of PS, PMMA and their blend.

In order to explore the impact of blending over thermal behaviour, the glass transition temperature (*T_g_*) and thermal degradation process were studied. The DSC plots of individual polymers and PS/PMMA blends are displayed in Figure 9. *T*_g_ values are displayed in Figure 10. The results demonstrated that *T*_g_ increased with increasing PS contents in the blend. The data reveals that the polymers were found to be miscible over the entire range of polymer compositions, represented by a single glass transition temperature, except for the blend with the 30/70 PS/PMMA composition shown by two separate glass transition temperatures, which indicates that blend in this ratio was immiscible [53] as demonstrated by viscosity and IR results. The thermo-gravimetric (TG) curves for the film formed from pure polymers and their blends in benzene are presented in Figure 11. The onset temperature (initial decomposition) is displayed in Figure 12. The onset temperature was found to be the lowest for PMMA and increased with an increasing amount of PS following the additive effect. It implies that PS is more stable as compared to that of PMMA. The rate of thermal degradation of these polymers and their blends was calculated from the slope of the TG curve for a particular temperature and provided by the software of the instrument is depicted in Figure 13. It is concluded that for PS/PMMA blend was in the order of 100/0 > 70/30 > 50/50 > 30/70 > 0/100 ratios. It indicates that although PS is more heat resistant, however, its rate of degradation is high, and this is attributed to the compact structure of PS as compared to the PMMA.

**Figure 14:**
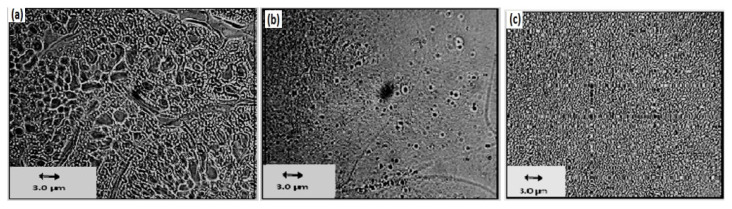
Optical micrograph of (a) 30/70 (PS/PMMA) blend film, (b) 50/50 (PS/PMMA) blend film and (c) 70/30 (PS/PMMA) blend film.

A comparison of the current and previously obtained quantities has been mentioned in Table 4. This comparison revealed somehow our results are quite close to the previous findings. To have a view of phase separation (omiscibilityty of blends), an optical micrograph of the PS/PMMA blending films was observed. Such images were taken of the dried film exhibited heterogeneous structure in each blend as shown in Figure 14(a–c). From such figures, a good trend has been observed, which clarifies that as the PS concentration is increasing from 50/50 onwards, the miscibility has increased and it is appearing as almost one phase.

## 5. Conclusion

PS and PMMA were blended using benzene as a solvent, and their viscosity, IR and thermal properties have also been investigated. The activation energy for the blends to flow was found to be very much dependent on blend composition, and interestingly was lower than those of pure polymers. The miscibility of polymers was calculated, measured, and analysed through many equations/techniques including Krigbaum and Wall equation and IR spectroscopy, and DSC. The results of the current work led to a conclusion that these polymers were found to be miscible at the 70/30 and the 50/50 ratios, and were found to be immiscible at the 30/70 ratios of PS/PMMA. The polymeric blends in the miscible range were more resistant to heat and exhibited a single melting temperature. The stability against thermal degradation or decomposition was reduced by the decrease in the miscibility of the polymers. Further, it was revealed that the higher thermal stability of PS could be related to its chemical structure, mainly because of the aromatic units, rather than its higher Mw.

## Figures and Tables

**Table 1:** Slope of the reduced viscosity versus concentration of PS/PMMA blends and individual solutions at different temperatures.

	bm			
PS/PMMA	20 °C	30 °C	40 °C	50 °C
**100/0**	0.258	0.292	0.308	0.352
**70/30**	0.43	0.442	0.476	0.506
**50/50**	0.357	0.379	0.409	0.412
**30/70**	0.128	0.149	0.159	0.163
**0/100**	0.104	0.118	0.122	0.138

**Table 2:** *Δ*b and *Δ*k_AB_ Values for the PS/PMMA blends at different temperatures.

PS/PMMA	20 °C		30 °C		40 °C		50 °C	
	Δb	Δk_AB_	Δb	Δk_AB_	Δb	Δk_AB_	Δb	Δk_AB_
70/30	0.3425	1.679	0.3445	2.554	0.2891	4.894	−0.0464	21.125
50/50	0.17475	1.125	0.21148	1.916	0.37695	3.718	0.0138	7.473
30/70	−0.06267	−0.46	−0.1777	−0.0172	−0.250397	−0.4376	−0.3807	−3.51

**Table 3:** *μ* and *α* Values for the PS/PMMA blends at different temperatures.

PS/PMMA	20°C		30°C		40°C		50°C	
	μ	α	μ	α	μ	α	μ	α
70/30	0.99	2.388	1.12	3.579	1.34	7.42	−2.24	4.639
50/50	0.496	5.495	0.69	23.54	1.75	28.57	0.19	12.14
30/70	−0.178	0.36	−0.58	0.86	−1.16	1.12	−18.36	−1.58

**Table 4:** A comparative view of the current and previous obtained quantities.

Analysis parameters	Comparison of the obtained results		Ref. #
	^*^Current work	Previous works	
FTIR analysis	PMMA indicated four absorption bands at 2949.75 cm^−1,^ 1723.42 cm^−1^, 1144.31 cm^−1^and 1239 cm^−1,^ that were attributed to C-H stretching. Polystyrene, the absorption bands which were visible at 3025.80 cm^−1^ showed C-H stretching.	The absorption bands of hydroxyl were observed at 3434.7 cm^−1^, 3500.8 cm^−1^, 3432 cm^−1^, 3439 cm^−1^, 3500.9 cm^−1^ 3497.7 cm^−1^ and 3478.9 cm^−1^ for compositions in PMMA/NC blend. PMMA indicated absorption bands at 3437.26 and at 3053.42 cm^−1^ which were assigned to O-H in carboxylic acid. Aromatic C-H stretch was found at 3053.42 cm^−1^. Two distinct bands appeared at 2978.4 & 2877.8 cm^−1^, the first band appeared from the asymmetrical (as) stretching mode in which two C–H bonds of the methyl group are extended.	[56] [54] [27]
Viscosity analysis	PMMA (reduced viscosity) (0.5~0.9) PS (reduced viscosity) (0.3~0.36)	PMMA (1.1 ~0.69) PS/PMMA/blend (0.0 ~0.75) PMMA (0.3009) PS (0.5274)	[56] [54] [27]
